# Experimental study on flow resistance characteristics of the uniform steam injection outflow control device

**DOI:** 10.3389/fchem.2023.1276691

**Published:** 2023-11-02

**Authors:** Qiuying Du, Mingzhong Li, Chenwei Liu

**Affiliations:** School of Petroleum Engineering, China University of Petroleum, Qingdao, China

**Keywords:** outflow control device, two-phase fluids, uniform steam injection, flow resistance characteristics, pressure drop

## Abstract

**Introduction:** Multi-point steam injection technology is a new completion method for heavy oil horizontal wells to solve the uneven distribution of the intake profile in the horizontal section. It is equipped with the flow control device to achieve the effect of balanced steam injection.

**Methods:** The steady-state experiment method was adopted; Considering the variable mass complex flow of the steam–liquid two-phase flow in the downhole flow device, the pressure loss of downhole tools through uniform steam injection with different steam–liquid compositions was tested, the influencing factors of the pressure drop were analyzed, and a more reliable pressure drop calculation method was established.

**Results:** The overflow pressure drop can be adjusted by changing the aperture, steam dryness, and fluid flow of the downhole outflow control device (OCD).

**Discussion:** By comparing the experimental and theoretical results, the calculation method of the overflow resistance of single-phase and steam–liquid two-phase fluids in OCD is given, and the error is within the usable range.

## 1 Introduction

In the process of steam injection in long horizontal wells, due to reservoir heterogeneity, heel–toe effect, and natural fractures in the reservoir, the suction profile of steam injection wells is difficult to maintain a balanced development, and steam channeling is easy to occur, and the heel end, high permeability interval, and fractures of wells are prone to premature water/steam. Due to low water/vapor viscosity, once water/gas coning occurs in the well, it will quickly form a channel at the coning point, thereby inhibiting oil production elsewhere (Mozaffari et al., 2013).

In order to reduce this non-uniformity, the flow control device, outflow control device (OCD), can be installed at the completion interval to inhibit the flow through the high-speed interval and generate additional pressure drop so as to increase the flow rate of the wellbore interval with high flow resistance, eliminate the non-uniform flow caused by the heel effect of the horizontal well and the non-uniformity of permeability, and ensure the uniform spread of the suction profile along the horizontal well (Parappilly, and Zhao, 2009; Gu et al., 2014a; Dong et al., 2014a; Dong et al., 2014b). The combination of a packer and OCD is usually used in production, which can achieve uniform oil drainage in heterogeneous reservoirs and maintain liquid output balance by limiting different oil production indexes in each section so as to delay bottom water coning, prolong anhydrous or bottom water oil production period, and improve oil and gas well production and recovery efficiency (Sun et al., 2018a; Sun et al., 2019). It is an advanced measure to stabilize oil and water and control water.

**FIGURE 1:**
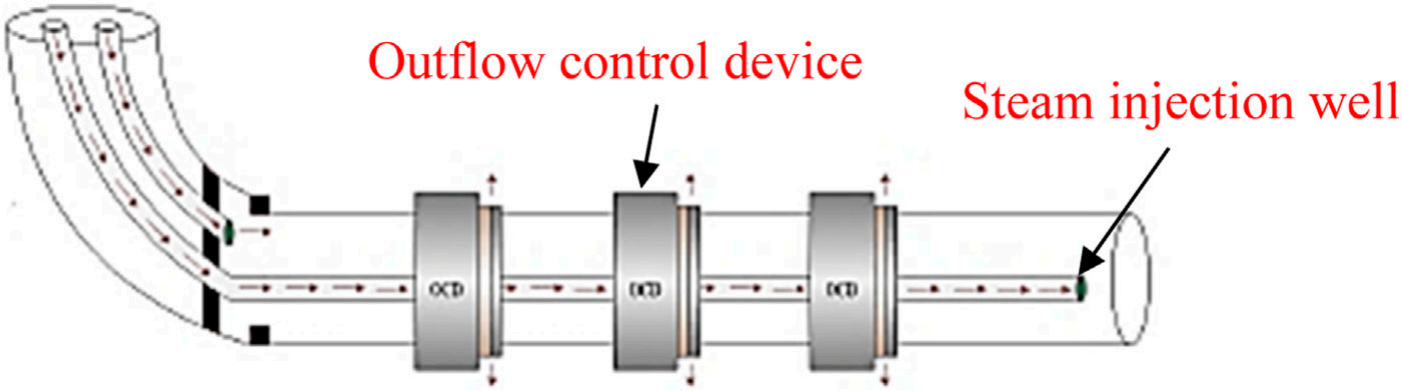
Schematic diagram of the outflow control device.

At present, a variety of OCDs has been developed in China. According to the mechanism of action, it can be divided into nozzle, spiral channel, and nozzle types (Huang et al., 2018a). The current limiting mechanism of nozzle-type OCD is that the throttle pressure drop is generated by the contraction of the flow channel when the fluid passes through the device (Kumar et al., 2010; Huang et al., 2018b). The spiral-channel-type OCD wraps one or more flow channels around the tubing to generate an additional pressure drop using friction (Rivas and Gates, 2018; Dong et al., 2020a; Dong et al., 2020b). Unlike the instantaneous pressure drop produced by the nozzle, this design produces a segmented pressure drop over a relatively long area, which is more resistant to the erosion and clogging of fluid particles during the drilling fluid cycle (Dong et al., 2020a). The nozzle-type OCD has a limited flow in a long nozzle. Compared with the nozzle-type OCD, owing to its longer spray irrigation, the resistance loss along the path is larger and the local resistance loss is smaller at the same strength (Hasan and Kabir, 1994; Hasan et al., 1998; Sun et al., 2018b). Therefore, compared with the three OCDs, the nozzle-type OCD is more resistant to the erosion and blockage of fluid particles (Hasan and Kabir, 2005; Caetano, 1985). Compared with the spiral-channel-type OCD, the resistance loss along the nozzle-type OCD plays a minor role in the pressure drop composition of the nozzle-type OCD, so the nozzle-type OCD is less sensitive to viscosity (Caetano et al., 1992; Gu et al., 2014b; Gu et al., 2015). The installation position of OCD on the horizontal steam injection well is shown in Figure 1.

Aiming at the completion characteristics of horizontal well OCD, this paper established a mathematical model of horizontal well variable mass flow under the condition of OCD installation based on the equivalent diameter model of the target well section, heterogeneous reservoir skin factor model, horizontal wellbore variable mass flow pressure drop model, and OCD pressure drop model by applying the principle of potential superposition. The effects of OCD aperture, steam dryness, and steam injection speed on the pressure drop were compared, and the OCD structure parameters and steam injection parameters were optimized. This method provides a new optimal control method for horizontal well uniform steam injection (Yin et al., 2019; Liu et al., 2022; Yin and Linga, 2019) and offers technical support for OCD completion optimization design and oil increase and water control in the horizontal well.

## 2 Experimental scheme

The main research content of this experiment is to measure the pressure drop of the fluid passing through the downhole flow control device and to establish a set of calculation methods of flow control resistance through the study of fluid resistance law so as to determine a reasonable OCD design method to achieve the purpose of uniform steam injection (Least et al., 2013; Yu et al., 2010). In order to study and analyze the flow resistance of different components of fluid that passes through the flow control device and analyze the influence of gas–liquid composition on the pressure drop, the pressure drop of single-phase liquid passing through the control device was first tested, and the pressure drop of fluid passing through the control device was analyzed by changing the flow rate, hole diameter, and fluid flow rate of the control device (Chesney et al., 2015). Then, the influence of the mixture flow rate and mass gas content on the pressure drop was analyzed by changing the mass gas content (Temizel et al., 2019).

### 2.1 Experimental apparatus and materials

**FIGURE 2:**
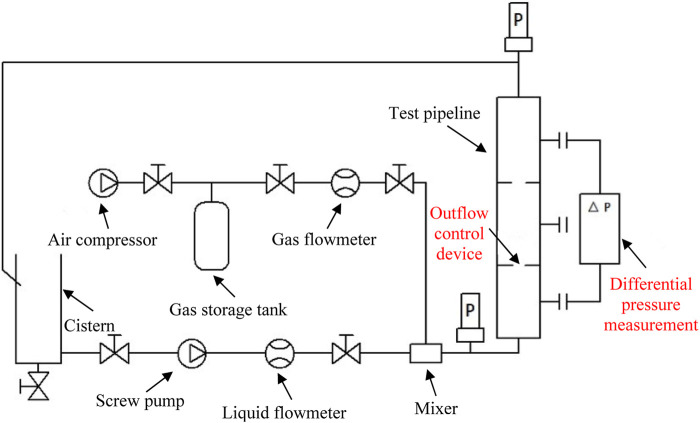
Schematic diagram of the downhole tool flow resistance test device.

The downhole tool flow resistance test system mainly comprises five parts: the gas supply system, liquid supply system, outflow control device (OCD) simulation system, measurement system (Gai et al., 2010), and data acquisition system (Figure 2). The fluid supply system consists of a liquid pump, inverter, air compressor, and gas pressure-reducing valve (Luo et al., 2015). The liquid pump uses a non-pulse screw pump. Its head measures 180 m, and the displacement range is 0–45 m^3^/h (Wang et al., 2016); the maximum pressure of the air compressor is 1.8 MPa, the maximum displacement is 5.1 m^3^/min, and the noise is 56 db; the gas storage tank volume is 1.5 m³, and working pressure is 0.8 MPa; it is made of Q345 material, equipped with a 1 MPa safety valve, and facilitates spray treatment. The gas-reducing valve is of YK43X type, with the maximum pressure of 1.6 MPa and output pressure range of 0.1–1 MPa. The accuracy of the pressure transmitter is 0.25% F.s, and the measuring ranges are 0–5 MPa and 0–1.6 MPa, respectively (Prakasa et al., 2019). The accuracy of the temperature sensor is class A, with a measuring range of 0°C–150°C, the flow sensor accuracy of 0.2%–0.5%, liquid flowmeter range of 0–16 m^3^/h, and gas flowmeter of 0–5.0 m^3^/min. The sensor can communicate with the computer through the A/D conversion board. The nozzle-type OCD is installed on the test completion string. The data acquisition and processing system comprises digital acquisition card, computer, and software. It carries out real-time data acquisition and post-processing and prepares original data reports, analysis reports, and curves, creates output database files, indicates the real-time display of control elements’ working status, and displays and prompts users to each stage of the workflow and pressure upper limit alarm.

### 2.2 Test procedure

Due to the nozzle-type flow control device used in the FluxRite™ completion string in the Mackay River oil sand, the fluid pressure drop through the flow control device was studied only under the conditions of single-phase liquid and high gas–liquid ratios (dryness).

**FIGURE 3:**
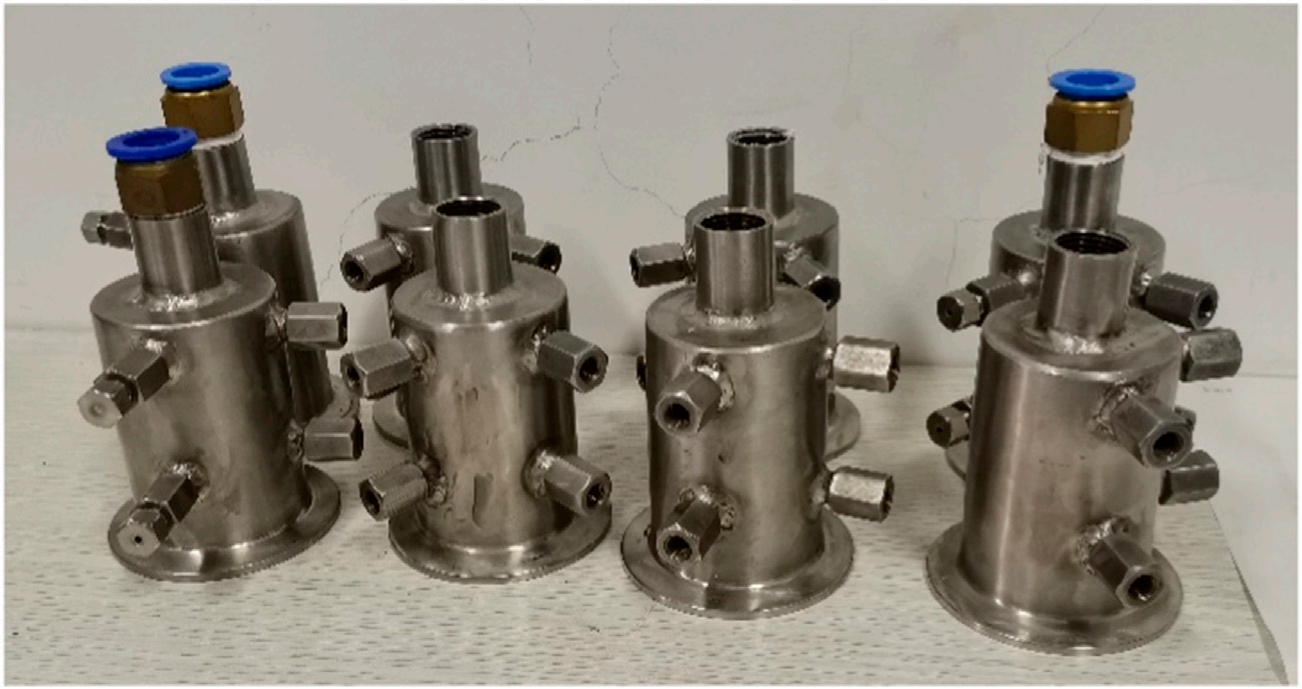
Flow control device simulator.

First, eight sizes of simulated nozzles were made according to the nozzle sizes of the FluxRite™ flow control device (2.5 mm, 4.0 mm), and four of them (2.5 mm, 3.0 mm, 4.0 mm, and 5.0 mm) were selected to conduct pressure difference loss experiments on single-phase liquid flow resistance (Figure 3). The test medium was tap water, with reference to the hot water injection flow of horizontal wells in Canada’s Mackay River block (0.3 m^3^/h), and the flow rates were 0.15 m^3^/h, 0.2 m^3^/h, 0.25 m^3^/h, 0.3 m^3^/h, 0.35 m^3^/h, and 0.4 m^3^/h. The pressure drop of the fluid through the simulated nozzle was recorded by the data acquisition system, and the relationship between the pressure drop and flow rate was analyzed.

Then, three nozzles of sizes 2.5 mm, 3.0 mm, and 4.0 mm were selected to carry out the resistance characteristic experiment of the gas–liquid two-phase flow through simulated nozzles. The gas–liquid mixture consisted of air and water, with reference to the flow rate of wet steam injection in horizontal wells in Canada’s Mackay River block (60 kg/h), and its mass flow rate was 50 kg/h, 55 kg/h, 60 kg/h, 65 kg/h, and 70 kg/h. Considering that the steam-assisted gravity drainage (SAGD) steam injection process is a relatively high-dryness flow process, the dryness of the gas–liquid mixture (that is, the mass content of the gas in the mixture) was set to 0.9, 0.8, and 0.7. The pressure difference of the fluid through the simulated nozzle is recorded by the data acquisition system, and the relationship between the pressure drop and the flow rate and the dryness of the mixture is analyzed.

### 2.3 Experimental procedure

The experimental procedure is as follows:

(1) Screw pump pumps the fluid in the liquid storage tank after suction and pressurization, with a certain flow and pressure through the flow control device.
(2) The liquid flow rate is measured by the liquid flowmeter, the size of the flow rate is controlled by the motor frequency converter, and the flow rate of the valve is recorded in real time.
(3) The pressure drop of the liquid through the flow control device is reflected and recorded in computer software by the differential pressure meter connected at both ends.
(4) The flow rate and pressure drop are monitored by the computer in real time, such that the pressure drop generated when fluid passes through the control device under a certain flow condition can be obtained.
(5) In the process of a single-phase liquid experiment, the liquid can be returned to the liquid storage tank through the pipeline for recycling.
(6) After the completion of the single-phase liquid experiment, start the compressor, adjust the gas-phase flow rate and liquid-phase flow rate, and when the flow is stable, record the pressure drop and dryness of the gas-liquid mixture in the outflow control device.

## 3 Results and discussion

### 3.1 Analysis of single-phase liquid flow resistance

**FIGURE 4:**
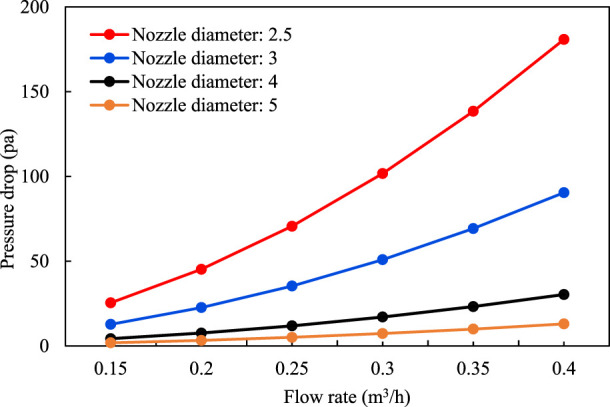
Single-phase liquid flow resistance calculation.

The pressure difference data of single-phase liquid flow resistance through the simulated nozzle are shown in Table 1 and the pressure drop curves under different nozzle diameters are shown in Figure 4. In the general literature, the formula for calculating the pressure drop of the fluid through the orifice plate or nozzle is used to obtain the throttle pressure difference when the fluid flows through the control valve:

$$\Delta p=\frac{1}{2}{\left(\frac{G_{l}}{C_{d}A_{p}\rho_{l}}\right)}^{2}\rho_{l},$$ (1)

**TABLE 1:** Experimental data of single-phase liquid flow resistance (kPa).

Flow rate (m^3^/h)		0.15	0.2	0.25	0.3	0.35	0.4
Nozzle diameter/mm	2.5	25.42	45.20	70.62	101.70	138.42	180.80
	3	12.72	22.61	35.32	50.87	69.23	90.43
	4	4.26	7.58	11.84	17.05	23.20	30.31
	5	1.83	3.25	5.07	7.30	9.94	12.98

where Δ*p* is the pressure difference before and after the throttle hole of the control valve, that is, the throttle pressure drop in Pa; *A*_p_ is the control valve orifice area in m^2^; and *C*_d_ is the flow coefficient without dimensionality. *G*_l_ is the steam mass flow rate in kg/s, and *ρ*_l_ is the fluid density in kg/m^3^. The value of *C*_d_ in common flow control valves ranges from 0.66 to 1.

According to the method of drawing the nozzle loss curve, the flow coefficient *C*_d_ of the fluid passing through the nozzle should not be a constant but a function of the hole diameter. According to the data of the pressure drop, flow rate, and hole diameter, the corresponding relationship between the flow coefficient *C*_d_ and hole diameter under different flow rates was obtained, and data fitting was carried out to obtain the fitting relationship between *C*_d_ and hole diameter:

$$C_{d}=1.3052d^{-0.11}=\frac{1.3052}{d^{0.11}}.$$ (2)

**FIGURE 5:**
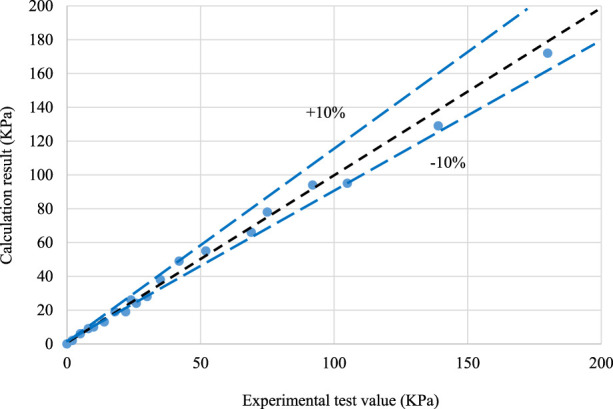
Fitting results of single-phase liquid flow resistance data.

The calculated results of the fitting relationship were compared with the experimental results (Figure 5). The comparison results show that the flow coefficient obtained by fitting can calculate the experimental data more accurately.

### 3.2 Flow resistance analysis of the gas–liquid two-phase fluid

The Pressure difference data of the gas–liquid mixture flow resistance through the simulated nozzle are shown in Tables 2–4.

**TABLE 2:** Pressure difference data of the gas–liquid mixture flow resistance through the 2.5-mm simulated nozzle.

Mass flow (kg/h)		50	55	60	65	70
Dryness	0.9	475.0	518.4	561.8	605.3	658.8
	0.8	269.9	287.1	312.3	339.6	365.4
	0.7	166.4	175.4	193.5	203.5	225.1

**TABLE 3:** Pressure difference data of the gas–liquid mixture flow resistance through the 3.0-mm simulated nozzle.

Mass flow (kg/h)		50	55	60	65	70
Dryness	0.9	331.2	367.3	398.4	429.5	465.6
	0.8	189.7	201.8	226.2	234.5	256.7
	0.7	114.6	126.1	134.9	149.7	165.2

**TABLE 4:** Pressure difference data of the gas–liquid mixture flow resistance through the 4.0-mm simulated nozzle.

Mass flow (kg/h)		50	55	60	65	70
Dryness	0.9	192.8	211.0	229.3	249.6	267.9
	0.8	109.5	115.7	126.9	136.2	144.4
	0.7	65.7	74.1	77.4	85.4	94.7

The calculation method of the pressure drop of gas–liquid mixture flow through the orifice plate proposed by Chisholm was used for data processing. Through the two-phase flow momentum equation analysis, the pressure drop of the fluid flowing through the orifice plate can be calculated as follows:

$$\Delta p_{\text{TP}}/\Delta p_{\text{LO}}=1+\frac{C}{X}+\frac{1}{X^{2}},$$ (3)

where *X* ^2^ is equal to △*p*_LO_/△*p*_GO_; △*p*_TP_ is the pressure drop of the gas–liquid two-phase mixture through the orifice plate in Pa; △*p*_LO_ and △*p*_GO_ are the pressure drop when the liquid and gas phases in the mixture, respectively, pass through the orifice plate alone in Pa; and *C* is a coefficient related to the pressure and slip ratio.

The following formula was used to calculate *X* ^2^:

$$X^{2}=\frac{{\left(1-x\right)}^{2}}{x^{2}}\frac{\rho_{G}}{\rho_{L}}.$$ (4)

In 1997, Chisholm suggested the following method for calculating the *C* values:

When *X* < 1, then

$$C={\left(\frac{\rho_{L}}{\rho_{G}}\right)}^{1/4}+{\left(\frac{\rho_{G}}{\rho_{L}}\right)}^{1/4}.$$ (5)

When *X* > 1, then

$$C={\left(\frac{\rho_{m}}{\rho_{G}}\right)}^{1/2}+{\left(\frac{\rho_{G}}{\rho_{m}}\right)}^{1/2}.$$ (6)

**FIGURE 6:**
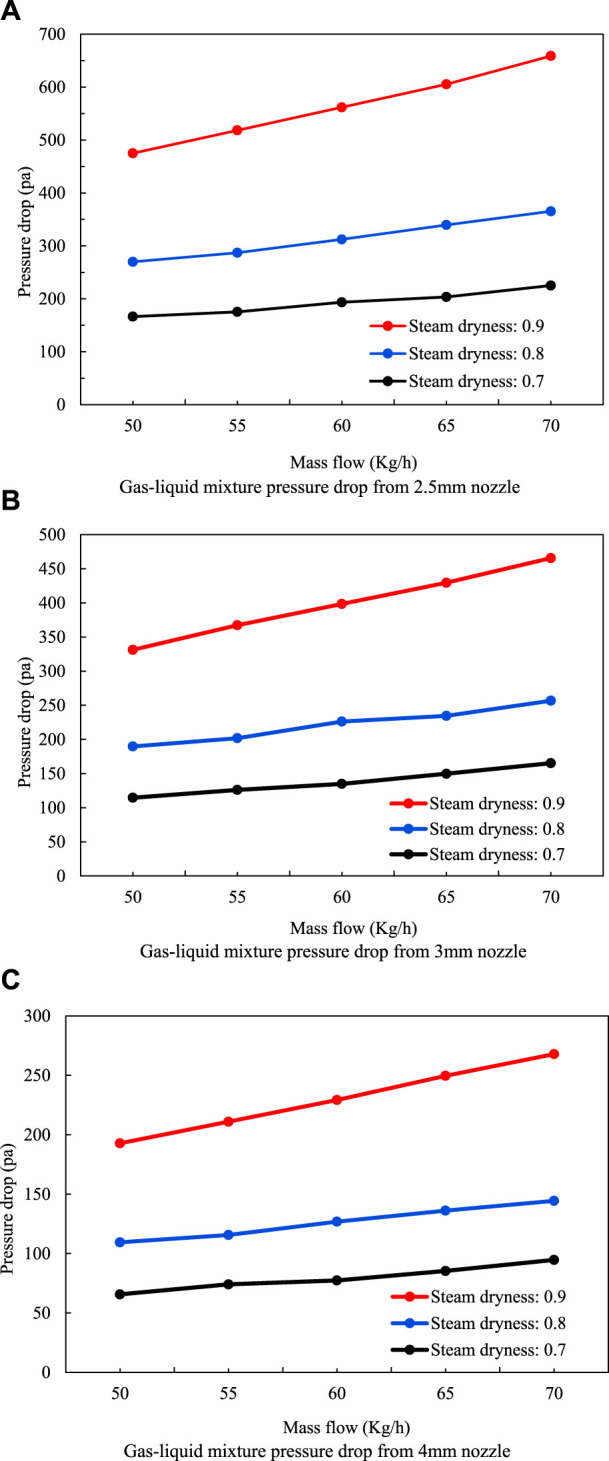
Calculation of flow resistance of the gas–liquid two-phase mixture. **(A)** Gas–liquid mixture pressure drop while using a 2.5-mm nozzle. **(B)** Gas–liquid mixture pressure drop while using a 3-mm nozzle. **(C)** Gas–liquid mixture pressure drop while using a 4-mm nozzle.

The calculation method proposed by Chisholm was used to calculate the pressure drop data of the gas–liquid mixture flow through the simulated nozzle. The pressure drop changes under the aperture of 2.5 mm, 3 mm, and 4 mm were obtained, as shown in the Figure 6, and the pressure drop decreased significantly when the nozzle size was increased. The greater the steam dryness, the greater the pressure drop and the better the effect of overflow resistance. The flow rate has little influence on the flow resistance effect, and the flow rate is positively correlated with the pressure drop.

**FIGURE 7:**
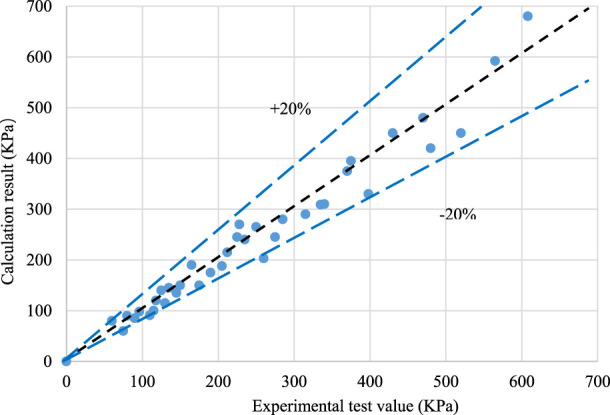
Experimental data calculation results of the gas–liquid mixture flow resistance through simulated nozzles.

The calculated results were compared with the experimental results (Figure 7). The comparison results show that the calculated values using Chisholm’s recommended method were consistent with the experimental results of the gas–liquid mixture, and the calculation error is less than ±20%.

### 3.3 Flow resistance analysis of the gas–liquid two-phase fluid with a large aperture

Since multi-stage OCD completion is usually used in the production process, that is, multiple OCDs are connected in series for steam injection at the same time, this paper designs a variety of large-aperture OCDs to meet production needs and installation on demand. Using the steam dryness of 0.9, steam injection pressure of 2 MPa, large aperture sizes of 20 mm, 50 mm, 80 mm, 110 mm, and 140 mm, the pressure drop and temperature change of steam through a valve under different apertures are calculated; the pressure drop calculation method of the gas–liquid two-phase flow is referred to formulas (3)–(6), and the heat calculation method is as follows:.

The temperature and pressure of saturated steam have a coupling relationship, which is given as follows:

$$T_{s}=210.2376p_{s}^{0.21}-30,$$ (7)

where *T*_*s*_ is the temperature of steam in °C and *p*_*s*_ is the pressure of steam in MPa.

The heat of the injected wet steam will reach the formation along the radial path through the inner tube of the insulated tubing, the insulation layer of the insulated tubing, the outer tube of the insulated tubing, and the annular space between the tubing and casing and the casing and cement ring. In this process, radial heat loss will be generated, which can be bounded by the outer edge of the cement ring. The former is the steady heat transfer, and the latter is the unsteady heat transfer. Convection heat transfer occurs when the wet steam flows through the inner wall of the insulated tubing, heat transfer occurs when it flows from the inner wall of the insulated tubing to the outer wall of the insulated tubing, convection heat transfer and heat radiation occur when it flows in the annular space between the tubing and casing, and heat transfer occurs when it flows in the annular space between the casing and cement ring; hence, the thermal resistance formula is expressed as follows:

$$\begin{array}{l} R=\frac{1}{2\pi h_{1}r_{1}}+\frac{1}{2\pi \lambda_{tub}}\ln\left(\frac{r_{2}}{r_{1}}\right)+\frac{1}{2\pi \lambda_{ins}}\ln\left(\frac{r_{3}}{r_{2}}\right)+\frac{1}{2\pi \lambda_{tub}}\ln\left(\frac{r_{4}}{r_{3}}\right)\\ +\frac{1}{2\pi \left(h_{c}+h_{r}\right)r_{4}}+\frac{1}{2\pi \lambda_{cas}}\ln\left(\frac{r_{co}}{r_{ci}}\right)+\frac{1}{2\pi \lambda_{cem}}\ln\left(\frac{r_{h}}{r_{co}}\right), \end{array}$$ (8)

where *h*_1_ is the convective heat transfer coefficient of wet steam in W/(m^2^·°C) and *r*1 is the inner tube radius of the insulated tubing in m. *λ*_tub_ is the thermal conductivity of the inner tube of the heat-insulated tubing in W/(m·°C); *r*_2_ is the inner and outer radius of the insulated tubing in m; *λ*_ins_ is the thermal conductivity of the insulation layer of the insulated tubing in W/(m·°C); *r*_3_ is the outer tube radius of the insulated tubing in m; *r*_4_ is the outer radius of the insulated tubing in m; *h*_c_ is the natural convection heat transfer coefficient in the annular space between the tubing and casing in W/(m^2^·°C); *h*_r_ is the radiant heat transfer coefficient in the annular space between the tubing and casing in W/(m^2^·°C); *λ*_cas_ is the thermal conductivity of the casing in W/(m·°C); *r*_co_ is the outer radius of the casing in m; *r*_ci_ is the tube radius in m; *λ*_cem_ is the thermal conductivity of the cement ring in W/(m·°C); and *r*_h_ is the outer radius of the cement ring in m.

The section of steam flowing through the nozzle is suddenly reduced, and the formula for calculating overflow resistance is given as follows:

$$F=\frac{P_{1}-P_{2}}{\gamma }+\frac{\alpha_{1}V_{1}^{2}}{2g}-\frac{\alpha_{2}V_{2}^{2}}{2g}-F_{1-2},$$ (9)

where *F* is the overflow resistance; *F*_1-2_ is the resistance loss along the path; *α*_1_ and *α*_2_ are the kinetic energy correction coefficients; *γ* is the fluid weight; and *V*_1_ and *V*_2_ are the average flow rates at the interface.

**FIGURE 8:**
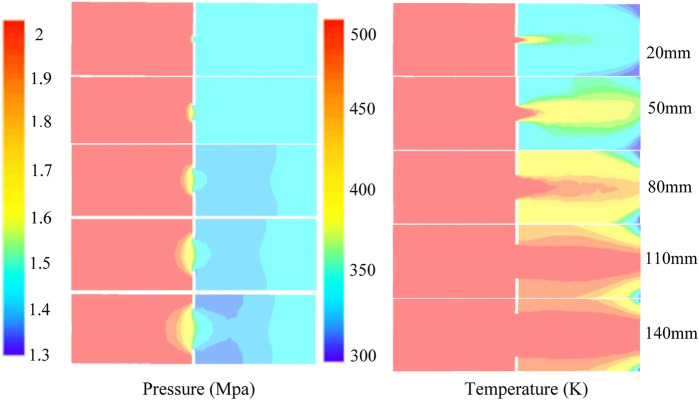
Distribution of the pressure field and temperature field of the steam flow in OCD.

**FIGURE 9:**
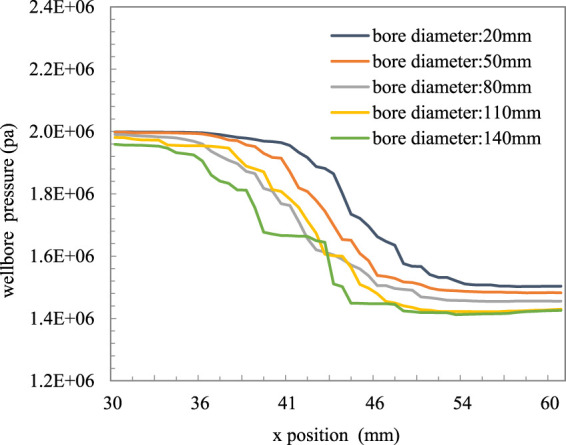
Relationship between pressure and pore size in OCD.

It can be observed from Figure 8 and Figure 9 that the pressure drop of the gas–liquid two-phase fluid varies greatly under different apertures. Because the mechanism of the local pressure drop of nozzle-type OCD depends on the minimum flow area, with the increase in the aperture, the pressure drop becomes smaller, the temperature range decreases, the heat transfer speed increases, and the heat loss decreases. When the pore diameter is greater than 110 mm, the effect of the steam pressure drop through the pore is not obvious, and the effect of OCD current limiting is worse.

## 4 Conclusion

(1) A simulation experimental device was established to carry out the flow resistance experiment of the single-phase liquid and gas–liquid mixture through the nozzle/orifice plate flow control device simulation system.
(2) Data analysis shows that the experimental data of the single-phase liquid flow can reliably be predicted using the formula of the liquid pressure drop through the orifice plate and the flow coefficient obtained by fitting the experimental data, and the prediction accuracy is less than ±10%; the calculation error of the experimental data of the gas–liquid mixture can be less than ±20% by using the method proposed by Chisholm.
(3) The factors affecting the flow resistance effect of the gas–liquid two-phase flow were compared and analyzed, and the overflow pressure drop effect under different pore sizes, steam dryness, and steam flow rates was simulated. It was concluded that the pore size had a significant effect on the pressure drop effect, and the pressure drop amplitude became smaller with the increase in the pore size. In order to achieve an obvious throttling effect, the maximum pore size should not exceed 80 mm; the higher the steam dryness, the more obvious the pressure drop effect is. The flow rate has little influence on the overflow resistance, and the overflow pressure difference becomes larger with the increase in the flow rate.

## Data Availability

The raw data supporting the conclusion of this article will be made available by the authors, without undue reservation.

## References

[B1] Caetano E. F. Shoham O. Brill J. P. (1992). Upward vertical two-phase flow through an annulus-Part I: single-phase friction factor, taylor bubble rise velocity, and flow pattern prediction. J. Energy Resour. Technol. 114, 1–13. 10.1115/1.2905917

[B2] Caetano E. F. (1985). Upward two-phase flow through an annulus. Tulsa, OK: University of Tulsa.

[B3] Chesney M. R. Felten F. Hallibuton H. S. Edlebeck J. (2015). Design, testing, and field performance of steam-injection flow-control devices for use in SAGD oil recovery. SPE, 174490. 10.2118/174490-MS

[B4] Dong X. H. Liu H. Q. Lu N. Wu K. Wang K. Chen Z. (2020a). Steam conformance along horizontal well with different well configurations of single tubing: an experimental and numerical investigation. SPE Prod. Operations 35, 549–563. 10.2118/195799-pa

[B5] Dong X. H. Liu H. Q. Pang Z. X. Wang C. Lu C. (2014b). Flow and heat transfer characteristics of multi-thermal fluid in a dual-string horizontal well. Numer. Heat. Transf. Part A Appl. 66, 185–204. 10.1080/10407782.2013.873255

[B6] Dong X. H. Liu H. Q. Zhai Y. Wang C. Chen Z. Liu D. (2020b). Experimental investigation on the steam injection profile along horizontal wellbore. Energy Rep. 6, 264–271. 10.1016/j.egyr.2020.01.005

[B7] Dong X. H. Liu H. Q. Zhang Z. X. Wang C. (2014a). The flow and heat transfer characteristics of multi-thermal fluid in horizontal wellbore coupled with flow in heavy oil reservoirs. J. Petroleum Sci. Eng. 122, 56–68. 10.1016/j.petrol.2014.05.015

[B8] Gai P. Du Y. Lu G. Li X. Li S. (2010). Uniform steam injection technology used in thermal horizontal wells. SPE, 130893. 10.2523/130893-MS

[B10] Gu H. Cheng L. Huang S. Zhang H. Lin M. Hu C. (2014b). A new semi-analytical model for predicting steam pressure and temperature in annuli. SPE, 170042. 10.2118/170042-ms

[B9] Gu H. Cheng L. S. Huang S. J. Du B. Hu C. (2014a). Prediction of thermophysical properties of saturated steam and wellbore heat losses in concentric dual-tubing steam injection wells. Energy 75, 419–429. 10.1016/j.energy.2014.07.091

[B11] Gu H. Cheng L. S. Huang S. J. Li B. Shen F. Fang W. (2015). Steam injection for heavy oil recovery: modeling of wellbore heat efficiency and analysis of steam injection performance. Energy Convers. Manag. 97, 166–177. 10.1016/j.enconman.2015.03.057

[B12] Hasan A. R. Kabir C. S. (2005). A simple model for annular two-phase flow in wellbores. SPE 95523. 10.2118/95523-PA

[B13] Hasan A. R. Kabir C. S. (1994). Aspects of wellbore heat transfer during two-phase flow. SPE Prod. Facil. 9, 211–216. 10.2118/22948-pa

[B14] Hasan A. R. Kabir C. S. Wang X. W. (1998). Wellbore two-phase flow and heat transfer during transient testing. SPE J. 3, 174–180. 10.2118/38946-pa

[B15] Huang S. J. Cao M. Xia Y. Chen X. Yang M. (2018b). Heat and mass transfer characteristics of steam in a horizontal wellbore with multi-point injection technique considering wellbore stock liquid. Int. J. Heat Mass Transf. 127, 949–958. 10.1016/j.ijheatmasstransfer.2018.07.136

[B16] Huang S. J. Xia Y. Xiong H. Liu H. Chen X. (2018a). A three-dimensional approach to model steam chamber expansion and production performance of SAGD process. Int. J. Heat Mass Transf. 127, 29–38. 10.1016/j.ijheatmasstransfer.2018.06.136

[B17] Kumar A. Oballa V. Card C. (2010). Fully-coupled wellbore design and optimization for thermal operations. SPE 137427. 10.2118/137427-MS

[B18] Least B. Greci S. Wilemon A. Ufford A. (2013). Autonomous ICD range 3B single-phase testing. SPE 166285. 10.2118/166285-MS

[B19] Liu X. J. Ren J. J. Chen D. Y. Yin Z. Y. (2022). Comparison of SDS and L-Methionine in promoting CO2 hydrate kinetics: implication for hydrate-based CO2 storage. Chem. Eng. J. 438, 135504. 10.1016/j.cej.2022.135504

[B20] Luo W. Li H. T. Wang Y. Q. Wang J. C. (2015). A new semi-analytical model for predicting the performance of horizontal wells completed by inflow control devices in bottom-water reservoirs. J. Nat. Gas Sci. Eng. 27, 1328–1339. 10.1016/j.jngse.2015.03.001

[B21] Mozaffari S. Nikookar M. Ehsani M. R. Sahranavard L. Roayaie E. Mohammadi A. H. (2013). Numerical modeling of steam injection in heavy oil reservoirs. Fuel 112, 185–192. 10.1016/j.fuel.2013.04.084

[B22] Parappilly R. Zhao L. (2009). SAGD with a longer wellbore. J. Can. Petroleum Technol. 48 (6), 71–77. 10.2118/09-06-71

[B23] Prakasa B. Muradov K. Davies D. (2019). Principles of rapid design of an inflow control device completion in homogeneous and heterogeneous reservoirs using type curves. J. Petroleum Sci. Eng. 176, 862–879. 10.1016/j.petrol.2019.01.104

[B24] Rivas D. A. Gates I. (2018). SAGD circulation phase: thermal efficiency evaluation of five wellbore completion designs in Lloydminster reservoir. SPE, 193357. 10.2118/193357-ms

[B25] Sun F. R. Yao Y. D. Li G. Z. Li X. Zhang T. Lu C. (2018b). An improved two-phase model for saturated steam flow in multi-point injection horizontal wells under steady-state injection condition. J. Petroleum Sci. Eng. 167, 844–856. 10.1016/j.petrol.2018.04.056

[B26] Sun F. R. Yao Y. D. Li G. Z. Liu W. (2019). A numerical model for wet steam circulating in horizontal wellbores during starting stage of the steam-assisted-gravity-drainage process. Heat Mass Transf. 55, 2209–2220. 10.1007/s00231-019-02564-7

[B27] Sun F. R. Yao Y. D. Li X. F. Li G. Liu Q. Han S. (2018a). Effect of friction work on key parameters of steam at different state in toe-point injection horizontal wellbores. J. Petroleum Sci. Eng. 164, 655–662. 10.1016/j.petrol.2018.01.062

[B28] Temizel C. Canbaz C. H. Palabiyik Y. Irani M. Balaji K. Ranjith R. (2019). Production optimization through intelligent wells in steam trapping in SAGD operations. SPE, 195361. 10.2118/195361-ms

[B29] Wang J. Liu H. Q. Liu Y. G. Jiao Y. Wu J. Kang A. (2016). Mechanism and sensitivity analysis of an inflow control devices (ICDs) for reducing water production in heterogeneous oil reservoir with bottom water. J. Petroleum Sci. Eng. 146, 971–982. 10.1016/j.petrol.2016.08.007

[B30] Yin Z. Y. Huang L L. Linga P. (2019). Effect of wellbore design on the production behaviour of methane hydrate-bearing sediments induced by depressurization. Appl. Energy 254, 113635. 10.1016/j.apenergy.2019.113635

[B31] Yin Z. Y. Linga P. (2019). Methane hydrates:A future clean energy resource. Chin. J. Chem. Eng. 27 (09), 2026–2036. 10.1016/j.cjche.2019.01.005

[B32] Yu T. T. Zhang H. Q. Li M. X. Sarica C. (2010). A mechanistic model for gas/liquid flow in upward vertical annuli. SPE Prod. Operations 25, 285–295. 10.2118/124181-pa

